# Job type, neighborhood prevalence, and risk of coronavirus disease 2019 (COVID-19) among healthcare workers in New York City

**DOI:** 10.1017/ice.2021.163

**Published:** 2021-04-15

**Authors:** Fran A. Ganz-Lord, Kathryn R. Segal

**Affiliations:** 1 Department of Medicine, Montefiore Medical Center, Bronx, New York; 2 Occupational Health Services COVID-19 Response, Montefiore Medical Center, Bronx, New York; 3 Albert Einstein College of Medicine, Bronx, New York

## Abstract

In this study, we compared the risk of coronavirus disease 2019 (COVID-19) between clinical and nonclinical healthcare workers (HCWs) while adjusting for home ZIP codes. Clinical HCWs did not have a higher risk of COVID-19, but living in higher-risk ZIP codes was associated with increased infection rates. However, environmental services workers showed increased risk of COVID-19.

As of January 14, 2021, an estimated 362,544 US healthcare workers (HCWs) had been infected with severe acute respiratory coronavirus virus 2 (SARS-CoV-2), the virus that causes coronavirus disease 2019 (COVID-19).^1^ Widespread availability of personal protective equipment (PPE), hand hygiene, and universal masking help mitigate the risk of SARS-CoV-2 infection,^2^ but questions remain about the risk for those in patient-facing roles. At the same time, communities of color, densely populated communities, and low-income communities have been shown to experience disproportionately high rates of infection.^3^ Although studies have begun to assess the impact of social factors, race, and ethnicity on infection among HCWs,^4^ further research is needed to understand how community prevalence impacts HCW risk. In this study, we compared SARS-CoV-2 infection between clinical and nonclinical employees of a New York City medical center while adjusting for ZIP-code–level risk.

## Methods

This observational, retrospective cohort study was conducted on HCWs at Montefiore Medical Center (MMC). Due to the lack of state ZIP-code–level comparison data, HCWs were included in this study only if they lived in New York City and had a polymerase chain reaction (PCR) test that was reported to the Occupational Health and Safety (OHS) Office between March 1 and September 30, 2020. ZIP-code–level rates of COVID-19 were determined using data published by the New York City Department of Health.^5^ ZIP codes that had case rates in the highest quartile were classified as very high risk (ie, >3,848.8 cases per 100,000 residents); those in the 50th–75th percentiles were classified as high risk (ie, between 3,228.1 and 3,848.8 cases per 100,000 residents); those in the 25th–50th percentiles were classified as medium risk (ie, between 2,280.9 and 3,228.1 cases per 100,000 residents); and those in the bottom quartile were classified as low risk (ie, <2,280.9 cases per 100,000 residents).

Multivariable logistic regression models were used to determine the association between clinical role and COVID-19 while adjusting for ZIP-code–level risk. Role type was abstracted from the OHS database, and HCWs were designated as “clinical” if their role required them to be in-person in the hospital or clinic with patient interaction. A second analysis was conducted to examine the association of different clinical roles on COVID-19 risk while adjusting for ZIP-code–level risk.

This study was approved by the Montefiore/Einstein Institutional Review Board. All analyses were performed using Stata version 11.2 software (StataCorp, College Station, TX).

## Results

Of 3,915 HCWs included in the final study population, 3,206 (81.9%) held clinical roles. Of all HCWs included in this study, 61.2% resided in a very high-risk ZIP code, 19.0% resided in a high-risk ZIP code, and 8.2% and 11.7% resided in a medium- or low-risk ZIP code.

In a logistic regression model that controlled for ZIP-code–level risk, we detected no significant difference in the odds of PCR positivity of clinical HCWs compared to nonclinical HCWs (OR, 1.05; 95% CI, 0.88–1.25). Residing in a very high-risk ZIP code (OR, 2.48; 95% CI, 1.93–3.18) or a high-risk ZIP code (OR, 2.39; 95% CI, 1.81–3.16) was significantly associated with higher odds of PCR positivity when compared to HCWs from low-risk ZIP codes.

In the secondary analysis, in comparison to non-clinical HCWs, environmental services (EVS) workers (OR, 2.16; 95% CI, 1.27–3.68) and clinical staff otherwise not categorized (OR, 1.29; 95% CI, 1.06–1.58) (see footnote of Table 1 for list of roles) were significantly associated with higher odds of PCR-positivity. Hospital staff physicians (OR, 0.42; 95% CI, 0.31–0.58) and attending physicians (OR, 0.67; 95% CI, 0.47–0.95) were significantly associated with lower odds. Residing in a very high-risk ZIP code (OR, 1.82; 95% CI, 1.38–2.39) or a high-risk ZIP code (OR, 1.86; 95% CI, 1.38–2.50) remained significantly associated with higher odds of PCR-positivity.

**Table 1.** The Association Between Job Type and ZIP-Code–Level Risk for COVID-19

Variable	Total	Positive	Negative	Results of Multivariable Logistic Regression Model With Clinical Versus Nonclinical Roles		Results of Multivariable Logistic Regression Model With Distinct Clinical Roles	
	(N = 3,915),	(N = 1,308),	(N = 2,607),	(N = 3,915)		(N = 3,915)	
	No. (%)	No. (%)	No. (%)	OR (95% CI)	*P* Value	OR (95% CI)	*P* Value
**Job title**							
Nonclinical	709 (18.1)	239 (18.3)	470 (18.0)	Ref	Ref	Ref	Ref
Clinical	3,206 (81.9)	1,069 (81.7)	2,137 (82.0)	1.05 (0.88–1.25)	.56		
Clerk/PSR	413 (10.6)	135 (10.3)	278 (10.7)			0.91 (0.71–1.18)	.49
EVS	60 (1.5)	32 (2.5)	28 (1.1)			**2.16 (1.27**–**3.68)**	**.004**
Nurse (LPN)	164 (4.2)	64 (4.9)	100 (3.8)			1.22 (0.86–1.73)	.27
Nurse (RN)	596 (15.2)	232 (17.7)	364 (14.0)			1.25 (0.99–1.56)	.06
NP or PA	103 (2.6)	34 (2.6)	69 (2.7)			1.11 (0.71–1.74)	.64
Other (clinical staff)^a^	1,040 (26.6)	408 (31.2)	632 (24.2)			**1.29 (1.06**–**1.58)**	**.01**
Physician: attending	285 (7.3)	57 (4.4)	228 (8.8)			**0.67 (0.47**–**0.95)**	**.03**
Physician: hospital staff	394 (10.1)	62 (4.7)	332 (12.7)			**0.42 (0.31**–**0.58)**	**<.001**
Respiratory therapist	36 (0.9)	11 (0.8)	25 (1.0)			0.85 (0.41–1.75)	.65
Security guard	34 (0.9)	11 (0.8)	23 (0.9)			0.90 (0.43–1.87)	.77
Social worker	81 (2.1)	23 (1.8)	58 (2.2)			0.84 (0.50–1.40)	.50
**ZIP code risk**							
Low	457 (11.7)	87 (6.7)	370 (14.2)	Ref	Ref	Ref	Ref
Medium	322 (8.2)	78 (6.0)	244 (9.4)	1.37 (0.97–1.93)	.08	1.12 (0.78–1.60)	.54
High	742 (19.0)	266 (20.3)	476 (18.3)	**2.39 (1.81**–**3.16)**	**<.001**	**1.86 (1.38**–**2.50)**	**<.001**
Very high	2,394 (61.2)	887 (67.1)	1,517 (58.2)	**2.48 (1.93**–**3.18)**	**<.001**	**1.82 (1.38**–**2.39)**	**<.001**

Note. OR, odds ratio; CI, confidence interval; PSR, patient service representative; EVS, environmental services; LPN, licensed practical nurse; RN, registered nurse; NP, nurse practitioner; PA, physician assistant. Data with statistical significance are shown in bold.

a Examples of roles included in the “other (clinical staff)” category: nurse attendants, transporters, phlebotomists, radiology technologists, and food delivery services.

## Discussion

In addition to potential exposures from SARS-CoV-2–positive patients and coworkers,^4^ HCWs are subject to infection risk in their home communities. Not surprisingly, our results show that HCW home ZIP-code risk was significantly associated with higher odds of PCR positivity. These findings support other studies that have shown community spread to be a significant risk for HCW infection.^6^ But our study also showed that when home ZIP code was accounted for, HCWs with clinical roles were not more likely to test positive for COVID-19 than HCWs in nonclinical roles. A previous study has already shown that symptomatic frontline workers did not have different rates of COVID-19 from nonfrontline workers.^7^ However, in our study, environmental services (EVS) workers had a higher risk of COVID-19 even when adjusting for risk from home ZIP code. The increased risk in EVS workers has been reported in previous studies,^8^ but to our knowledge, none of these adjusted for community prevalence. We noted that some EVS workers hang their masks on their cart and change frequently based on the assigned isolation status of the room, which causes frequent touching of masks. In addition, observations revealed lower hand hygiene between rooms by EVS workers than by nurses and physicians. MMC responded to this data with a change to consistent PPE protocols, data sharing, and programs to re-emphasize procedures around processes like donning and doffing PPE. An aggregated designation of “other clinical role” was also associated with a higher risk of COVID-19, but due to the disparity in roles in this category, we are not able to draw conclusions for this group.

In our study, when we adjusted for ZIP code, physicians were at lower risk of contracting COVID-19 than nonclinical HCWs. This finding could, in part, be due to the fact that we were unable to separate physicians who were doing telemedicine from those working in-person during the initial spring surge. However, by including data through the summer, it is likely that most physicians included spent some time in-person with patients. In addition, our testing results were limited to those who tested through or reported results to the OHS. Physicians could theoretically have had more access to ordering tests and may have been more likely to bypass these pathways. But our findings still raise interesting questions about physician behavior during the pandemic. Understanding what is driving the lower risk could identify practices that could be expanded to other HCWs.

A limitation of this study is potential generalizability to other medical centers whose HCWs may not live in urban and high-prevalence communities. MMC is located in the Bronx, which has consistently shown some of the highest case rates in the country.^9^ More than 60% of the HCWs in this study come from ZIP codes deemed very high risk based on New York City data. Importantly, the ZIP codes that were in the highest quartiles of case rates are more racially and ethnically diverse and are lower income, on average.^10^ We were also limited to only HCWs who live in New York City due to the lack of availability of ZIP-code–level COVID-19 data outside the city. Lastly, race, ethnicity, and comorbidity data were not available for this observational and retrospective study, and we were unable to include a comparison group of non-HCWs. Future analyses should include these critical factors.

The findings of this study suggest that current PPE protocols are protecting clinical HCWs as intended. However, continued vigilance outside of work is critical. Our study supports the need for additional protections for EVS workers. Hospitals should consider protocols that do not have differential PPE requirements based on the isolation status of the patient room and a closer look for additional areas of potential vulnerabilities.

## References

[1] CDC COVID data tracker: cases and deaths among healthcare personnel. Centers for Disease Control and Prevention website. https://covid.cdc.gov/covid-data-tracker/?CDC_AA_refVal=https%3A%2F%2Fwww.cdc.gov%2Fcoronavirus%2F2019-ncov%2Fcases-updates%2Fcases-in-us.html#health-care-personnel. Published 2021. Accessed January 14, 2021.

[2] Wang X , Ferro EG , Zhou G , Hashimoto D , Bhatt DL. Association between universal masking in a healthcare system and SARS-CoV-2 positivity among healthcare workers. JAMA 2020;324:703–704. 32663246 10.1001/jama.2020.12897 PMC7362190

[3] Whittle RS , Diaz-Artiles A. An ecological study of socioeconomic predictors in detection of COVID-19 cases across neighborhoods in New York City. BMC Med 2020;18:271. 32883276 10.1186/s12916-020-01731-6 PMC7471585

[4] Nguyen LH , Drew DA , Graham MS , et al. Risk of COVID-19 among frontline healthcare workers and the general community: a prospective cohort study. Lancet Public Health 2020;5(9):e475–e483. 32745512 10.1016/S2468-2667(20)30164-X PMC7491202

[5] New York City COVID-19 data. NYC Health website. https://www1.nyc.gov/site/doh/covid/covid-19-data.page. Published 2020. Accessed November 20, 2020.

[6] Rosser JI , Röltgen K , Dymock M , et al. SARS-CoV-2 seroprevalence in healthcare personnel in northern California early in the COVID-19 pandemic. *Infect Control Hosp Epidemiol* 2020. doi: 10.1017/ice.2020.1358. PMC7783083 33292895

[7] Mani NS , Budak JZ , Lan KF , et al. Prevalence of COVID-19 infection and outcomes among symptomatic healthcare workers in Seattle, Washington. Clin Infect Dis 2020;71:2702–2707. 32548613 10.1093/cid/ciaa761 PMC7337651

[8] Eyre DW , Lumley SF , O’Donnell D , et al. Differential occupational risks to healthcare workers from SARS-CoV-2 observed during a prospective observational study. eLife 2020;9:e60675. 32820721 10.7554/eLife.60675 PMC7486122

[9] COVID-19 US cases by county by the Center for Systems Science and Engineering (CSSE) at Johns Hopkins University. Johns Hopkins University website. https://coronavirus.jhu.ed/s-map. Published 2020. Accessed November 23, 2020.

[10] Social determinants of health database (beta version). Agency for Healthcare Research and Quality website. https://www.ahrq.gov/sdoh/data-analytics/sdoh-data.html. Accessed March 22, 2021.

